# Pandemic and prejudice: Results from a national survey experiment

**DOI:** 10.1371/journal.pone.0265437

**Published:** 2022-04-13

**Authors:** Neeraj Kaushal, Yao Lu, Xiaoning Huang

**Affiliations:** 1 School of Social Work, Columbia University, New York, NY, United States of America; 2 Department of Sociology, Columbia University, New York, NY, United States of America; 3 Feinberg School of Medicine, Northwestern University, Chicago, IL, United States of America; Free University of Bozen-Bolzano, ITALY

## Abstract

Do health and economic shocks exacerbate prejudice towards racial/ethnic minority groups? We investigate this question in the wake of the COVID-19 pandemic by collecting nationally representative survey data with an embedded experiment. Results show that priming COVID-19 salience has an immediate impact: compared to the control group, respondents in the treatment group reported increased prejudice towards East Asian and Hispanic colleagues. East Asians in the treatment group faced higher prejudicial responses from Americans living in counties with higher COVID-19 infections and those who lost jobs due to COVID-19, and fewer prejudicial responses in counties with a higher concentration of Asians. These results point to the salience of COVID-19 fueled health and economic insecurities in shaping prejudicial attitudes, specifically towards East Asians. County-level socioeconomic factors did not moderate the increased prejudicial attitudes toward Hispanics in the workplace. These findings highlight a dimension of prejudice, intensified during the pandemic, which has been largely underreported and therefore missing from the current discourse on this important topic.

## Introduction

Do health and economic shocks exacerbate prejudice towards racial/ethnic minorities? We investigate this question in the wake of the COVID-19 pandemic that has cratered the world economy, upended lives and livelihoods, caused massive social disruption and alienation, stoked widespread public health fears and risks, and cost, as of this writing, more than five million lives globally. Specifically, we conducted a nationally representative survey with an embedded experiment in a hypothetical workplace setting to examine if COVID-19 salience fueled prejudices towards racial/ethnic minorities.

Workplace discrimination affects not merely the economic opportunities of minorities but also their physical and mental health, productivity, career advancement, economic progress, and integration with the society at large [1–4]. It can further alienate minorities and sow seeds of distrust that can have long-term impacts spilling across generations.

Our research speaks to reports of rising instances of anti-Asian racism, particularly towards Chinese Americans, since the start of the COVID-19 pandemic [5–7]. Several organizations, including the Stop AAPI Hate website, have been documenting increases in hate crime cases against Asians in the past year [7]. These reports, based on self-reported instances of hate crime, though informative, suffer from reporting bias in that the less empowered, who are often the most vulnerable, may fear reporting harassment and hate crime. The less visible, everyday incidents remain underreported [8, 9]. In particular, reporting of workplace discrimination is far less common for fear of retribution from the employer and fellow workers. The vast majority of existing reports have focused on discrimination in the social domain, most often perpetrated by strangers. We have limited understanding of whether COVID-19 has fueled workplace discrimination against minority groups. Further, most reports are specific to Asians, and do not provide data on attitudes towards other minority groups, who may also have been subject to discrimination under these strenuous health and economic circumstances.

We systematically investigate prejudicial attitudes towards various racial/ethnic groups by collecting original nationally-representative survey data (N = 5,000; working age sample [19–64 years old] = 3,837) in August 2020, when the pandemic had spread across the United States and was still raging. A unique feature of our research is that we measure prejudice using self-reported attitudes elicited through a vignette experiment. This strategy mitigates potential reporting and social desirability biases [10, 11].

Specifically, our research adopts a two-layer experimental design. The first layer of the experiment involved randomly exposing half the respondents (treatment group) to a short text on the state of COVID-19 (as of August 2020), followed by a set of questions on how COVID-19 impacted employment, earnings, and health of the respondents and their families. We refer to this as *COVID-19 information treatment*. These respondents then completed a vignette experiment and other survey questions. The remaining respondents (control group), first completed the vignette experiment and other survey questions and, at the very end of the survey, answered the questions on how COVID-19 impacted their lives. The Materials and Methods section provides detailed description of the study design and the Section 1 and S1 Fig in S1 File describe the COVID-19 information treatment.

In the vignette, which is the second layer of the experiment, we introduced each respondent to a hypothetical employee in a hypothetical workplace setting (Section 1 and S2 Fig in S1 File). The vignette was identical in all respects except for the name of the employee, which was randomized to signal the ethno-racial group of the employee (henceforth, referred to as the *race/ethnicity treatment*). Upon reading the vignette, the survey asked each respondent to rank their preference for having the hypothetical individual as a colleague, staff member, or boss, on a scale of 0 to 10 (extremely unlikely to extremely likely). We examined the attitudes of respondents towards five different ethnoracial groups: Whites, Blacks, Hispanics, East Asians, and South Asians.

We expect the COVID-19 information treatment (a reminder of COVID-19 and its impact on the respondents and their families) to exacerbate health and economic insecurities of the treatment group and in turn activate or increase ill-feelings and prejudices towards minorities. Insecurities emanating from the disease threat may result in defensive responses in the form of unwillingness to interact with, or work along with, coworkers feared to be conduits of the disease [12, 13]. We expect such prejudices and fears to be particularly strong towards East Asians, whose ancestry is associated with the origin of the COVID-19 virus and who were the subject of racist political rhetoric that framed the coronavirus as the “Chinese virus” or “Asian flu”.

Further, we expect COVID-19 fueled workplace prejudice to extend beyond East Asians towards other minority groups that are generally perceived as foreign (e.g., Hispanic in the United States) and "undeservedly" encroaching on limited national resources during the pandemic (e.g., unemployment benefits, economic relief, and limited job opportunities during COVID-19). Media and political rhetoric on COVID-19 has increased xenophobic sentiments by offering xenophobic explanations and solutions for the pandemic. An increase in prejudicial sentiment towards other minority groups will serve as evidence that COVID-19 has elevated xenophobic sentiments more broadly.

Since the outbreak of COVID-19, a number of papers, based on surveys of selective populations, have found rising instances of discrimination towards minorities, generally in the public arena and perpetrated by strangers. A summary of the published research is in Section 2 in S1 File. One recent study used nationally representative survey experiment and found evidence of COVID-19 triggered prejudice and discriminatory intent towards Asians and Hispanics in a social setting. But so far there is little systematic investigation on discrimination in the economic arena [14].

Our research builds on these studies and makes several contributions. First, ours is the first study to investigate whether COVID-19 fueled workplace prejudice among fellow coworkers. The focus of most previous studies and media reports has largely been hate crime in the public and social arena. We use a nationally representative survey experimental design that strengthens causal inference and permits generalizability of our findings.

Second, we examine attitudes towards all major ethno-racial groups, not just Asians. We expect the prejudices fueled by COVID-19 to be pronounced for East Asians but also to extend beyond East Asians. Note that we compare attitudes of respondents in the treatment and control groups towards hypothetical colleagues from the *same* racial/ethnic groups. Thus, any discrimination on account of assumptions about qualities of workers from different racial/ethnic groups (statistical discrimination) would be canceled out in the treatment versus control group comparison for that race/ethnicity. Further, the vignette experiment included identical information on the qualities of the hypothetical coworker (SI1; e.g., proficient and perseverant) to minimize discrimination on account of perceived quality.

Three, our study examines the factors and conditions that aggravate or mitigate the negative effects of COVID-19 on attitudes towards minorities. Circumstances that condition public attitudes towards minorities are not well understood. Individual characteristics and experiences in the health, social, economic, and political domains may be important moderators. Further, variation across localities in the spread of COVID-19 and economic hardship offer a unique opportunity to test the heterogeneity of pandemic-triggered discrimination. Focusing on variations across these individual and contextual factors allows us to understand multiple dimensions of discrimination.

Briefly, we find that priming COVID-19 salience had an immediate impact: compared to the control group, respondents in the treatment group reported increased prejudicial response towards accepting East Asians as colleagues and supervisors. COVID-19 treatment also increased prejudicial response towards Hispanic colleagues, supervisors, and staff. The treatment effect was more pronounced in reducing extreme positive attitudes toward Hispanics coworkers; but for East Asian colleagues and staff, the treatment both increased extreme negative attitudes and extreme positive attitudes. We do not find evidence of differential response towards White, Black, and South Asian co-workers (colleagues, staff, or supervisors) between the treatment and control groups.

We identify several important moderators. COVID-19 treatment resulted in East Asians facing higher prejudicial responses from Americans living in counties with higher COVID-19 infections and from Americans who lost jobs due to COVID-19, but fewer prejudicial responses from individuals living in counties with a higher concentration of Asians. County-level variation in COVID-19 infections or other county level characteristics did not moderate the treatment effects on prejudicial attitudes towards Hispanics. Further analysis shows that individuals with COVID-19 in their network were more favorable to accepting East Asian colleagues and Hispanic supervisors, which could suggest that the lived experience about the pandemic in one’s network increased empathy towards minorities. We note that having someone in the network with COVID-19 is different from the COVID-19 information treatment, which is a reminder of the socioeconomic and health shocks that COVID has caused. COVID-infections have been relatively mild in a majority of cases. Having had COVID therefore may have modest or, as we find, more empathetic impact on attitudes towards minorities.

## Materials and methods

### Research design

We conducted a nationally representative online survey with experiments of 5,000 American adults between August 13 and August 31, 2020, of which 3,837 were working age (19–64). The focus of this study is working-age Americans who are most likely to relate to workplace scenarios. The survey captured a period when the threat presented by the pandemic was striking: the novel coronavirus was on the rise and had spread widely across all US states. YouGov, a reputed survey agency used by many academic researchers to study public opinion, administered the online survey. YouGov maintains a large panel of respondents and routinely collects data on their basic demographic and socioeconomic characteristics, geographic location, and political affiliation. Its sampling framework allows for drawing nationally representative samples. Existing research has shown that YouGov samples are of high quality and representative of the target population [15].

We conducted a pilot online survey during July 14–16, 2020 and used the findings from the pilot to refine our questionnaire and conduct a power analysis. The power analysis suggested that a sample size of 3,800 would allow us to detect small effects at α = 0.05 with over 80% power.

#### Participant consent

Columbia University’s Institutional Review Board (IRB) approved the study. All survey participants signed an online written consent form to their participation in the survey. The study did not include minors. We introduced the survey to all respondents as a “Survey of Life Circumstances and Public Opinion of United States’ Residents”, which is the title of the survey.

YouGov used a sample-matching methodology, which is ideally suited for online surveys [16]. First, a random sample was drawn from the target population (US adults) based on the 2018 American Community Survey (i.e., target sample). For each member of the target sample, one or more matching members from the YouGov panel were selected. Matching was accomplished using a large set of variables available for both the target population and the YouGov panel, including age, sex, race, education, employment, and region of residence [17, 18]. The matched cases were weighted to the sampling frame using propensity scores to ensure representative-ness of the US adult population. YouGov’s sample matching procedure replaces nonrespondents with similar respondents (based on observed basic demographic, SES, and region characteristics). These procedures resulted in a sample that matched the profile of the target sample and is thus considered nationally representative.

### Experimental design

Our survey experiment design had two primary layers. First, the survey began with randomly assigning half the respondents (treatment group) to what we call a COVID information treatment. This involved exposing the treatment group to a short paragraph on the current state of COVID-19 (in August 2020; S1 Fig in S1 File), followed by a set of questions on how COVID-19 impacted employment, earnings, and health of respondents and their families. The aim of the COVID-19 information treatment was to increase the salience of the pandemic for the treatment group by priming them to think about the pandemic and its impacts globally as well as on their personal lives. The remaining respondents (control group) completed the survey questions on their opinions about coworkers first, and then, at the tail end of the survey, viewed the COVID-related paragraph and answered the set of questions on the impact of COVID-19. Our analysis confirmed that respondents exposed to the two conditions did not exhibit systematic differences in observable characteristics (S4 Table in S1 File).

The second layer of our survey experiment was a vignette experiment, in which each respondent was presented with a hypothetical situation to elicit their prejudice and discriminatory intent in workplace settings. The vignette described a hypothetical employee (S2 Fig in S1 File) with certain characteristics. All respondents read identical vignettes except for the name of the hypothetical employee, which was expected to signal the ethno-racial categorization of the employee (S5 Table in S1 File with the names [e.g., Brian Chen; Mr. Chen] randomly assigned). Upon reading the vignette, the respondent was asked to rank their preference for having the hypothetical individual as a colleague, supervisor, or staff (subordinate), on a scale from 0 to 10 (extremely likely to extremely unlikely). The order of the questions (colleague, subordinate, or boss) was randomized. We refer to this second layer experimental randomization as the racial/ethnic treatment. This vignette experiment, designed to study discrimination in the workplace, is adapted from Berdahl and Min [19].

We randomized names in the vignettes so that each respondent was presented with only one name (versus each respondent evaluating workers of different race/ethnicity) to reduce social desirability bias [11]. We used seven different names (S5 Table in S1 File) to represent the following ethno-racial groups: Whites, Blacks, Hispanics, East Asian Americans, East Asian immigrants, South Asian Americans, and South Asian immigrants. We combine East/South Asian immigrants and Americans to increase sample size. This is also because separate analysis does not reveal systematic differences between the two groups of East Asians and South Asians.

We used only male names to avoid difference in preference on account of the gender of the coworker. We carefully selected names by examining population-based racial/ethnic naming patterns in the US Census data and the New York State birth record data. These names have been validated in previous research [20, 21].

Respondent participation in the survey was voluntary. Each respondent signed a consent form. The survey was designed to take 15 minutes. The median time of completion was 15.5 minutes. To ensure quality, we applied a standard attention screener and dropped a small number of individuals who completed the survey too quickly or skipped too many questions. The survey had a response rate of 60.3% (completed interviews among all the invitations) and a cooperation rate of 91.4% (completed interviews among those who started the survey). The completion rate was not significantly different between the treatment and control groups.

**Fig 1:**
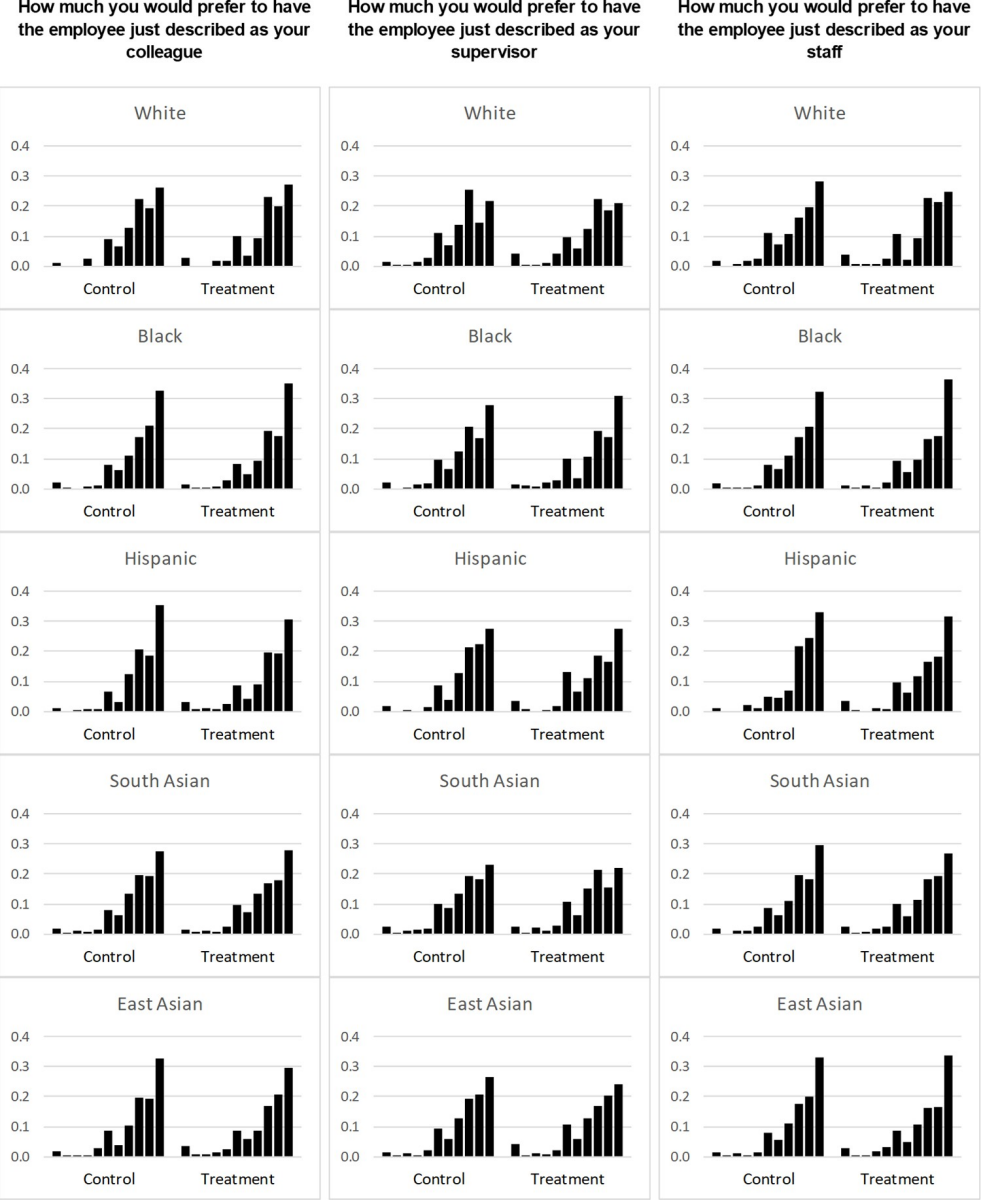
Responses in the coworker experiment.

**Fig 2:**
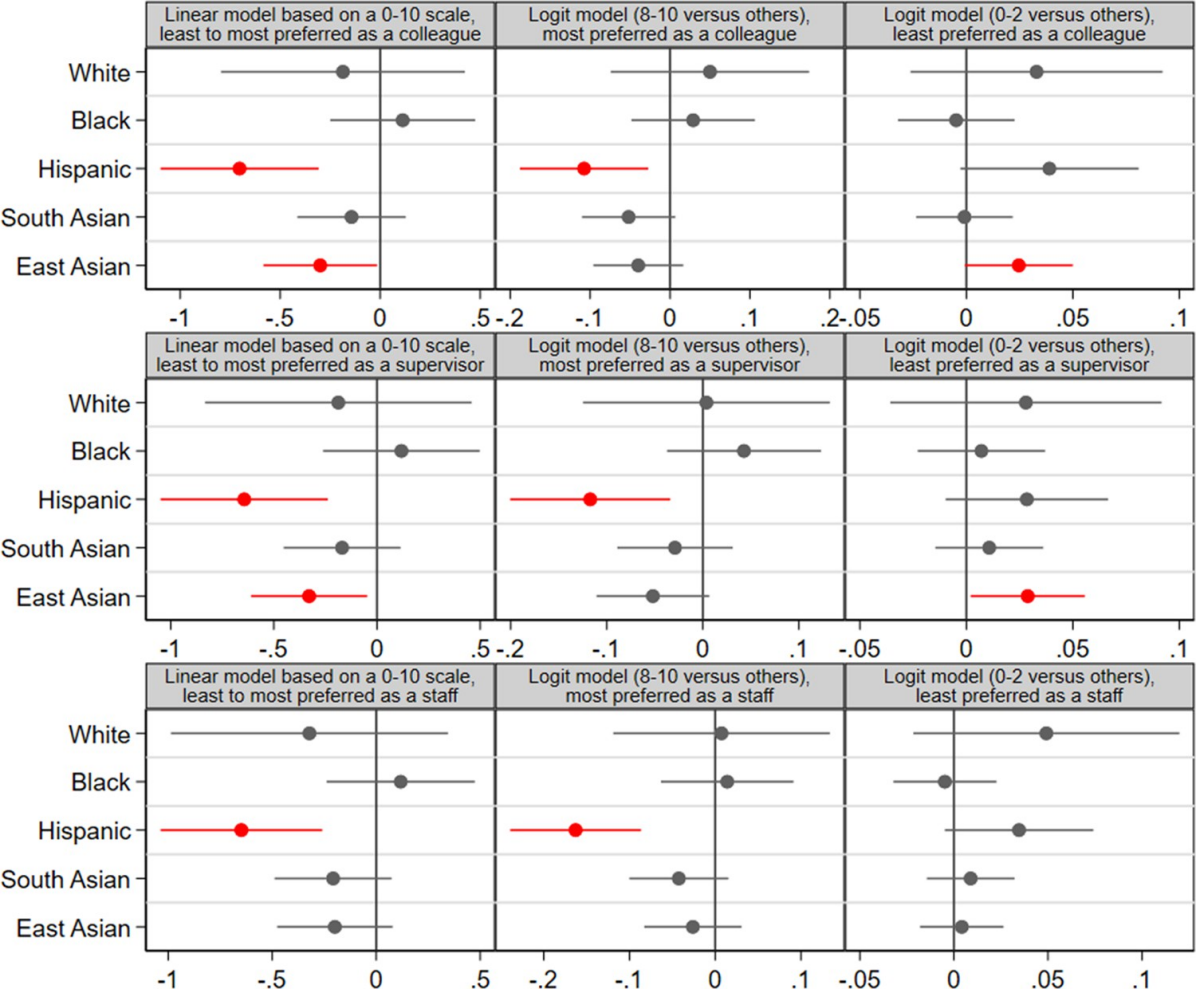
COVID-19 information treatment effect on attitudes towards hypothetical coworkers by race/ethnicity.

Our experiment pertains to workplace discrimination. Because workplace threats from COVID-19 and its consequences, whether economic and health-related, are more real for the working age population, we restrict the sample of our analysis to respondents aged 19–64 years. We first study workplace attitudes of all working age Americans (S1 Table in S1 File), and then excluding individuals belonging to the race signaled in the vignette (Figs 1 and 2 in the main manuscript). For robustness, we also conducted the analysis adjusted for pre-treatment characteristics (S2 Table in S1 File) and with the full sample of all adult Americans (S3 Table in S1 File).

Our survey included a rich set of variables on respondents’ demographic characteristics, namely, age, education, gender, marital status, race, nativity, employment status, political identity, and state of residence, which we controlled for in regression analyses. Our survey also includes information on respondent’s postal code, which allows us to construct county-level variables. County-level data on unemployment rate are from the Bureau of Labor Statistics [22]; COVID-cases and COVID-linked deaths from CDC and state health departments [23]; political affiliation from McGovern et al. 2020 [24]; and ethnic composition data from the US Census Bureau [25].

## Results

### Overall treatment effects

Fig 1 presents the raw responses for the three questions (namely preference for a colleague, a supervisor, and a staff) by the race/ethnicity of the hypothetical worker and by the treatment and control groups. The figure provides a few broad patterns. The proportion of respondents giving low scores (least favorable) to hypothetical coworkers (score 0–2) is relatively modest, ranging between 1% and 5% across groups. But there is substantial variation in the proportion with higher scores (most favorable) across hypothetical workers’ race/ethnicity and between treatment and control groups.

Fig 2 and Table 1 presents estimates of the treatment effect, showing that priming COVID-19 salience increased prejudicial response towards East Asians and Hispanics coworkers. Compared to the control group, individuals in the treatment group (exposed to a reminder of COVID-19 and its impact before the survey) expressed greater unwillingness to work with, or to be supervised by, East Asians. Further, the treatment group was less willing to work with, to be supervised by, or accept Hispanics as their staff members (subordinates), compared to the control group.

**Table 1:** COVID-19 information treatment effects on workplace prejudice (sample excludes signaled race).

		How much you would prefer to have the employee just described as your colleague			How much you would prefer to have the employee just described as your supervisor			How much you would prefer to have the employee just described as your staff		
	N	Linear (0–10)	Approve (8/10)	Oppose (0/2)	Linear (0–10)	Approve (8/10)	Oppose (0/2)	Linear (0–10)	Approve (8/10)	Oppose (0/2)
**White**	223	-0.19	0.05	0.03	-0.19	0.00	0.03	-0.32	0.01	0.05
		(0.31)	(0.06)	(0.03)	(0.33)	(0.07)	(0.03)	(0.34)	(0.06)	(0.04)
**Black**	536	0.11	0.03	-0.00	0.12	0.04	0.01	0.12	0.01	-0.00
		(0.18)	(0.04)	(0.01)	(0.19)	(0.04)	(0.02)	(0.18)	(0.04)	(0.01)
**Hispanic**	456	-0.70*	-0.11*	0.04^†^	-0.64*	-0.12*	0.03	-0.65*	-0.16*	0.03^†^
		(0.20)	(0.04)	(0.02)	(0.21)	(0.04)	(0.02)	(0.20)	(0.04)	(0.02)
**South Asian**	1019	-0.14	-0.05^†^	-0.00	-0.17	-0.03	0.01	-0.21	-0.04	0.01
		(0.14)	(0.03)	(0.01)	(0.14)	(0.03)	(0.01)	(0.14)	(0.03)	(0.01)
**East Asian**	1016	-0.30*	-0.04	0.02^†^	-0.33*	-0.05^†^	0.03*	-0.20	-0.03	0.00
		(0.14)	(0.03)	(0.01)	(0.14)	(0.03)	(0.01)	(0.14)	(0.03)	(0.01)

Notes: Sample is restricted to working age (19–64) respondents. The samples exclude respondents of the same race/ethnicity as the hypothetical coworker. Each cell in the table is based on a different regression and provides estimates of COVID information treatment effects. For each question, the first column presents coefficients based on linear regressions. The second and third columns present average marginal effects based on logistic regressions. Row-headings describe hypothetical co-worker’s race/ethnicity. Standard errors are in parenthesis.

^†^ p<0.1

* p<0.05.

The treatment effect was more striking in reducing extreme positive attitudes toward Hispanics coworkers; but for East Asian colleagues and staff, the treatment both increased extreme negative attitudes as well as reduced extreme positive attitudes (Table 1). The analyses in Table 1 exclude the racial/ethnic group signaled in the vignette experiment. For instance, when studying attitudes towards Hispanics, we excluded Hispanic respondents from the sample and when studying attitudes towards East Asians we excluded East Asian respondents from the sample. These results hold in robustness checks using samples that include the racial/ethnic group signaled in the vignette experiment (S1 Table, Section 3 in S1 File), in models that control for pre-treatment covariates (S2 Table in S1 File), and when the sample includes all Americans—working age adults and the elderly (S3 Table in S1 File). S6 Table in S1 File presents the full model on attitudes towards colleagues that include all control variables.

The differences in co-worker preferences towards White, Black, and South Asian co-workers (colleague, staff member, or supervisor) between the treatment and control groups are modest and statistically insignificant. Whites and Blacks are generally perceived as most “American” and are likely less vulnerable to pandemic-related discrimination, particularly if it is rooted in xenophobia. In models including (S1 Table in S1 File) and excluding (S2 Table in S1 File) respondents of the signal race, the results were similar, again showing no treatment effect for Black and White co-workers. This suggests that our estimates are primarily not affected by in-group bias.

Lack of statistically significant effects in the case of South Asian co-workers is at first surprising. Further investigation suggests a possible explanation: the signaling of South Asian names is less strong than other names. The proportion of respondents that passed the manipulation check is lower for South Asian names (60%) than for the other groups (Whites 85%; Blacks 70%; Hispanics 83%; and East Asians 76%). This corroborates previous research that shows many Americans do not identify South Asians as Asians (26). We conducted a sensitivity analysis for the South Asian signal race by excluding respondents who failed the manipulation check. The estimated effects were in the expected direction, suggesting that COVID-19 increased prejudice towards this group, but the estimates were statistically insignificant, likely due to the notably reduced sample size.

Next, we explore several potential moderation factors for the East Asian and Hispanic coworkers.

#### Exposure to pandemic health risks and prejudice

First, we examine if discrimination in the workplace is moderated by differences in exposure and vulnerability to the pandemic. Specifically, we study if individuals who were exposed to greater disease risks (i.e., living in counties with above average COVID-19 cases) or had direct experience with COVID-19 (i.e., had someone in their network infected with COVID-19) exhibited more negative attitudes.

Results (Table 2.) show that the COVID-19 information treatment effect towards East Asians is greater among those who lived in counties with higher than national COVID-19 infections. At the individual level, however, COVID-19 treatment does not trigger differential response towards East Asians or Hispanics between those who themselves (or family members) contracted COVID-19 and those who did not. This finding suggests that COVID-19 triggered discrimination towards East Asians had largely emanated from the fear of the pandemic (which is likely higher in counties with higher than national COVID-19 infections), and not from actual personal experience of COVID-19.

**Table 2:** COVID-19 information treatment effect on attitudes towards hypothetical coworkers, by respondent’s exposure to health risks.

	Hispanic Coworker			East Asian Coworker		
	Colleague	Supervisor	Staff	Colleague	Supervisor	Staff
**Panel 1: Individual Exposure to COVID**						
** COVID information treatment**	-0.62*	-0.44	-0.52^†^	-0.27	-0.28	-0.13
	(0.29)	(0.30)	(0.28)	(0.22)	(0.22)	(0.21)
** Had COVID cases in network**	0.22	0.66*	0.15	0.50*	0.54*	0.49*
	(0.25)	(0.26)	(0.24)	(0.18)	(0.19)	(0.19)
** Treatment × Had COVID**	-0.22	-0.46	-0.27	-0.11	-0.14	-0.22
** cases in network**	(0.40)	(0.41)	(0.38)	(0.28)	(0.30)	(0.28)
**Panel 2: Contextual Health Risk of COVID**						
** COVID information treatment**	-0.76*	-0.60*	-0.69*	0.01	-0.05	-0.08
	(0.29)	(0.28)	(0.27)	(0.18)	(0.18)	(0.17)
** County had more COVID cases**	0.28	0.56*	0.23	0.13	0.08	0.03
** than national per capita**	(0.25)	(0.26)	(0.27)	(0.21)	(0.21)	(0.21)
** Treatment × County had more**	0.13	-0.08	0.10	-0.85*	-0.77*	-0.42
** cases than national per capita**	(0.37)	(0.37)	(0.37)	(0.31)	(0.31)	(0.28)

Notes: Sample is restricted to working age (19–64) respondents. The samples exclude respondents of the same race/ethnicity as the hypothetical coworker. Each column within each panel in the table is based on a linear regression. Outcomes on coworker preferences are measured on a scale of 0 to 10 (higher number indicating greater preference). All regression models control for the order of questions asked, respondent’s age, gender, race, education, marital status, family income, log value of population in county of residency, and region of residency. The top-row column headings list the race/ethnicity of the hypothetical co-worker in the vignette. Standard errors are in parenthesis and clustered at county level.

^†^ p<0.1

* p<0.05.

An interesting finding in Table 2. (panel 1) is that individuals with COVID-19 in their network were more favorable to accepting East Asian colleagues and Hispanic supervisors, which likely suggests that the lived experience about the pandemic in one’s network increased empathy towards minorities. We emphasize that having someone in network with COVID-19 is very different from the COVID-19 information treatment, which is a reminder of the socioeconomic and health shocks that COVID has caused.

#### Economic recession and prejudice

We examine if the economic fallout of the pandemic that left millions without jobs and triggered extreme economic insecurity moderated discrimination in the work place. Our survey collected detailed data on whether a respondent was working at the time of the interview, whether they were not working due to COVID, or whether they were not working for other reasons. We investigate if COVID-treatment triggered a differential response towards accepting minority persons as colleagues (supervisors or staff) across respondents in each of these economic circumstances.

Results (Table 3) show that the COVID-19 treatment effect on accepting East Asians as staff members is stronger for those who personally experienced unemployment due to COVID-19. The COVID-19 information treatment did not elicit a different response from individuals who lived in high versus low unemployment counties. This finding suggests that COVID-19 triggered discrimination towards East Asians may have emanated from personal experience of economic insecurity due to COVID-19. For Hispanics, the interaction coefficients, while negative, are not statistically significant, suggesting that COVID information treatment effects were pervasive. Note that our analysis is based on a single wave of data, and therefore, we are not able to include more parsimonious models with county fixed effects. Our findings could be confounded with other geographic factors we do not adequately control for (such as those correlated with unemployment).

**Table 3:** COVID-19 information treatment effect on attitudes towards hypothetical coworkers by respondent’s exposure to economic risks.

	Hispanic coworker			East Asian coworker		
	Colleague	Supervisor	Staff	Colleague	Supervisor	Staff
**Panel 1: Individual Employment Status**						
** COVID information treatment**	-0.48^†^	-0.41	-0.47^†^	-0.32^†^	-0.30	-0.16
	(0.26)	(0.26)	(0.25)	(0.19)	(0.21)	(0.18)
** Not working due to COVID**	-0.02	0.23	-0.18	0.29	0.37	0.32
** **(Compared to currently working)	(0.42)	(0.41)	(0.43)	(0.29)	(0.31)	(0.27)
** Not working due to other reasons**	0.35	0.57^†^	0.23	-0.07	-0.03	-0.07
** **(Compared to currently working)	(0.31)	(0.31)	(0.30)	(0.23)	(0.22)	(0.22)
** Treatment × Not working**	-0.78	-0.57	-0.40	-0.71^†^	-0.76^†^	-0.96*
** due to COVID**	(0.64)	(0.64)	(0.69)	(0.41)	(0.41)	(0.39)
** Treatment × Not working**	-0.53	-0.64	-0.49	0.28	0.17	0.18
** due to other reasons**	(0.44)	(0.45)	(0.43)	(0.33)	(0.34)	(0.33)
**Panel 2: County Unemployment Rate**						
** COVID Information Treatment**	-0.74*	-0.67*	-0.67*	-0.36*	-0.35*	-0.36*
	(0.24)	(0.23)	(0.24)	(0.16)	(0.17)	(0.15)
** County unemployment rate**	0.08	0.14	0.20	-0.57*	-0.46^†^	-0.59*
** greater than national average**	(0.27)	(0.30)	(0.27)	(0.27)	(0.26)	(0.27)
** Treatment × County unemployment**	0.05	0.05	-0.00	0.05	-0.09	0.43
** rate greater than national average**	(0.39)	(0.41)	(0.35)	(0.36)	(0.37)	(0.33)

Notes: Sample is restricted to working age (19–64) respondents. The samples exclude respondents of the same race/ethnicity as the hypothetical coworker. Each column within each panel in the table is based on a linear regression. Outcomes on coworker preferences are measured on a scale of 0 to 10 (higher number indicating greater preference). All regression models control for the order of questions asked, respondent’s age, gender, race, education, marital status, family income, log value of population in county of residency, and region of residency. The top-row column headings list the race/ethnicity of the hypothetical co-worker in the vignette. Standard errors are in parenthesis and clustered at county level.

^†^ p<0.1

* p<0.05.

#### Diversity and prejudice

We examine if discrimination in the workplace is moderated by the social environment in which Americans live. Results in Table 4 show that the proportion of East-Asians in the county lowered COVID-treatment triggered discriminatory response towards East Asian coworkers. As mentioned above, our analysis is based on a single wave of data, and therefore, we are not able to include more parsimonious models with county fixed effects. Our finding could be confounded with other geographic factors correlated with ethnic density.

**Table 4:** COVID-19 information treatment effect on attitudes towards hypothetical coworkers, by respondent’s social contact and level of diversity in county of residence.

	Hispanic coworker			East Asian coworker		
	Colleague	Supervisor	Staff	Colleague	Supervisor	Staff
**Panel 1: Individual Social Contact**						
** COVID information treatment**	-1.09*	-0.96*	-0.99*	-0.46*	-0.47*	-0.34^†^
	(0.36)	(0.36)	(0.35)	(0.20)	(0.20)	(0.19)
** Had some contact with**	0.17	0.47^†^	0.35	0.36*	0.37*	0.26
** the ethnic group in question**	(0.24)	(0.27)	(0.24)	(0.18)	(0.18)	(0.18)
** Treatment × Had some contact with**	0.65	0.57	0.60	0.32	0.28	0.23
** the ethnic group in question**	(0.42)	(0.43)	(0.43)	(0.27)	(0.28)	(0.28)
**Panel 2: Contextual Level of Diversity**						
** COVID information treatment**	-0.95*	-0.77*	-0.87*	-0.60*	-0.48*	-0.48*
	(0.33)	(0.32)	(0.31)	(0.20)	(0.20)	(0.19)
** Share of the ethnic**	0.19	0.48	0.78	-0.18	0.52	-2.17
** group in county**	(0.85)	(0.93)	(0.80)	(1.44)	(1.55)	(1.58)
** Treatment × Share of the**	1.55	0.81	1.46	4.56*	1.96	3.83*
** ethnic group in county**	(1.35)	(1.29)	(1.21)	(1.76)	(2.12)	(1.88)

Notes: Sample is restricted to working age (19–64) respondents. The samples exclude respondents of the same race/ethnicity as the hypothetical coworker. Each column within each panel in the table is based on a linear regression. Outcomes on coworker preferences are measured on a scale of 0 to 10 (higher number indicating greater preference). All regression models control for the order of questions asked, respondent’s age, gender, race, education, marital status, family income, log value of population in county of residency, and region of residency. The top-row column headings list the race/ethnicity of the hypothetical co-worker in the vignette. Standard errors are in parenthesis and clustered at county level.

^†^ p<0.1 *

p<0.05.

Having personal contact with East Asians before the pandemic, however, did not moderate the effect of COVID-19 treatment. Moderating effects of living in counties with large Hispanic population or prior social contact on the COVID-19 treatment effect towards Hispanics were not significant.

#### Increasing incidents of racism during Politics and prejudice

COVID-19 have generally been blamed on President Trump, who used racially charged terms to describe the pandemic [26]. We test if workplace discrimination varies with the political affiliation of respondents or degree of progressiveness in their counties of residence. We specifically compare respondents across a number of categories: (i) respondent is a self-identified Democrat, Republican, or Independent/other; and (iii) respondents in counties with different levels of conservatism (measured by the county’s proportion of Trump votes in the 2016 presidential election). We do not find any evidence that COVID-19 treatment impacted discriminatory response of respondents depending on the level of progressiveness in their county of residence or on their personal political affiliations (Table 5).

**Table 5:** COVID-19 information treatment effect on attitudes towards hypothetical coworkers, by respondent’s political orientation and environment.

	Hispanic coworker			East Asian coworker		
	Colleague	Supervisor	Staff	Colleague	Supervisor	Staff
**Panel 1: Individual Political Orientation**						
** COVID information treatment**	-0.73*	-0.61^†^	-0.52^†^	-0.59*	-0.53*	-0.45^†^
	(0.32)	(0.33)	(0.31)	(0.25)	(0.24)	(0.23)
** Democrats**	-0.17	-0.14	-0.06	0.21	0.27	0.35^†^
** **(Compared to Independent/Others)	(0.28)	(0.33)	(0.28)	(0.23)	(0.22)	(0.21)
** Republicans**	-0.15	-0.21	-0.25	-0.12	-0.20	-0.10
** **(Compared to Independent/Others)	(0.32)	(0.32)	(0.31)	(0.28)	(0.29)	(0.26)
** Treatment x Democrats**	0.16	0.24	0.05	0.41	0.24	0.30
	(0.48)	(0.50)	(0.45)	(0.33)	(0.32)	(0.31)
** Treatment × Republicans**	-0.20	-0.51	-0.58	0.44	0.36	0.41
	(0.51)	(0.52)	(0.52)	(0.38)	(0.40)	(0.38)
**Panel 2: County Political Environment**						
** COVID information treatment**	-1.15*	-1.11*	-1.00*	-0.86*	-0.87^†^	-0.54
	(0.49)	(0.47)	(0.48)	(0.44)	(0.45)	(0.39)
** Share of Trump votes in 2016**	-0.06	-0.51	0.41	-0.84	-0.07	-0.19
	(0.82)	(0.86)	(0.79)	(0.69)	(0.68)	(0.68)
** Treatment × Share of**	0.97	1.04	0.75	1.14	1.12	0.63
** Trump votes in 2016**	(1.06)	(1.02)	(1.00)	(0.90)	(0.92)	(0.82)

Notes: Sample is restricted to working age (19–64) respondents. The samples exclude respondents of the same race/ethnicity as the hypothetical coworker. Each column within each panel in the table is based on a linear regression. Outcomes on coworker preferences are measured on a scale of 0 to 10 (higher number indicating greater preference). All regression models control for the order of questions asked, respondent’s age, gender, race, education, marital status, family income, log value of population in county of residency, and region of residency. The top-row column headings list the race/ethnicity of the hypothetical co-worker in the vignette. Standard errors are in parenthesis and clustered at county level.

^†^ p<0.1 *

p<0.05.

#### US-China trade war and prejudice

We also study if prejudice towards East Asians is linked with US-China trade war that peaked in 2019 with China and the US imposing retaliatory tariffs towards each other. Extant research shows that competition from China exposed Americans to greater job churning and lowered lifetime income [27]. We use export-supported jobs under tariff retaliation as a proportion of county population as a measure of the economic threat from US-China trade war [28]. We study if the COVID-19 treatment had a different impact on Americans living in counties differentially affected by the trade war. Results suggest that Americans living in counties more adversely affected by US-China trade war expressed heightened discriminatory response towards East Asian supervisors. However, we find no evidence that COVID-19 salience impacted the response of the treatment group differently across counties that were differentially impacted by the trade war (Table 6).

**Table 6:** COVID-19 information treatment effect on attitudes towards hypothetical coworkers by exposure to retaliatory tariffs in respondent’s county of residence.

	Hispanic coworker			East Asian coworkers		
	Colleague	Supervisor	Staff	Colleague	Supervisor	Staff
**COVID information treatment**	-6.84^†^	-6.49	-7.58*	-5.82^†^	-6.45**	-6.78*
	(4.06)	(4.53)	(3.72)	(3.24)	(3.19)	(3.45)
**Share of total export-supported**	3.29	4.81	3.78	4.89	3.62	2.13
**jobs under retaliation in county**	(5.45)	(5.09)	(4.75)	(4.29)	(4.21)	(4.30)
**Treatment × Share of total export-**	0.65	0.31	1.05	-0.22	0.65	0.35
**supported jobs under retaliation**	(0.78)	(0.80)	(0.71)	(0.65)	(0.64)	(0.59)

Notes: Sample is restricted to working age (19–64) respondents. The samples exclude respondents of the same race/ethnicity as the hypothetical coworker. Each column within each panel in the table is based on a linear regression. Outcomes on coworker preferences are measured on a scale of 0 to 10 (higher number indicating greater preference). All regression models control for the order of questions asked, respondent’s age, gender, race, education, marital status, family income, log value of population in county of residency, county’s share of trump votes in 2016, and region of residency. The top-row column headings list the race/ethnicity of the hypothetical co-worker in the vignette. Standard errors are in parenthesis and clustered at county level.

^†^ p<0.1

* p<0.05.

#### Age, sex, and prejudice

Our primary analysis is based on working age adults. In additional analysis, we tested if increase in discrimination towards East Asian and Hispanic colleagues that we observe varies by working age (19–64) and 65+ populations (S7 Table in S1 File). The results show that the treatment effects were largely concentrated among working-age adults in case of attitudes towards Hispanics. In case of East Asians, the interaction between the COVID-19 treatment and age is statistically insignificant, suggesting a more prevalent treatment effect towards East Asians. We also estimated regressions allowing the effect of COVID-information treatment to vary by gender. We find no evidence that the treatment affected men and women differently (p-value of the interaction term = 0.28–0.42).

## Discussion

The COVID-19 pandemic has brought the biggest public health calamity since the 1918 Influenza pandemic and the most severe economic decline since the Great Depression of 1929. In this paper, we systematically examine how the pandemic has shaped attitudes towards minorities in the workplace in the United States. Our research speaks to the torrent of violence and hate crimes against minority groups, especially East Asians, since the start of the pandemic, and suggests that the increased discrimination generally reported in the social settings has likely spilled over to the economic settings. Our nationally representative survey experiment in a hypothetical workplace setting showed that priming COVID-19 salience exaggerated discriminatory response towards accepting East Asians and Hispanics as colleagues. We also find a significant treatment effect on attitudes towards accepting East Asians as supervisors, and accepting Hispanics as supervisors and staff members. The treatment effect was particularly pronounced in increasing extreme negative attitudes than in decreasing extreme positive attitudes towards East Asians, whereas the treatment had a more pronounced effect in reducing extreme positive attitudes towards Hispanics.

Our research validates findings from a recent study that found the salience of COVID-19 treatment exaggerating prejudice and discriminatory intent towards accepting Asian and Hispanics in social interactions (i.e., in search of roommates [14]). At the same time, we find noteworthy differences between the workplace (economic) and the roommate (social) settings. Unlike in the social domain where anti-Asian prejudice was pervasive, prejudice against East Asians in the workplace varied across locations and situations. First, findings from the survey experiment show that East Asians faced higher discriminatory responses from the treatment group living in counties with higher COVID-19 infections. Second, members of the treatment group who lost jobs due to COVID-19 were more likely to express prejudicial responses than those who did not. Third, East Asians faced fewer prejudicial responses from the treatment group who lived in counties with a higher concentration of Asians. Overall, evidence points to the salience of COVID-19 fueled health and economic insecurities in shaping prejudicial attitudes towards East Asians.

We find that the COVID-19 treatment triggered exaggerated negative attitudes towards accepting East Asians as colleagues and supervisors, but not as staff. This could be because most people cannot relate to a scenario in which they make hiring decisions, whereas most working-age adults can conceive of, and indeed operate in, situations of working and interacting with colleagues and supervisors. COVID-19 triggered negative attitudes towards Hispanics, on the other hand, were generalized towards all three groups of co-workers: colleagues, supervisors, and staff. While our analysis does not allow us to speculate the reason for this finding, it points towards rising xenophobia during COVID-19, which contributes to the development of anti-Hispanic attitudes, even towards a less relatable hiring scenario.

We do not find evidence of differential response towards White, Black, and South Asian co-workers (colleagues, staff, or supervisors) between the treatment and control groups. Whites and Blacks are generally perceived as most “American” and may thus be less vulnerable to pandemic-related discrimination, particularly if it is rooted in xenophobia or targeting the group typically associated with COVID-19. The absence of a COVID-19 treatment effect towards Blacks could also be because of the countering effect of rising empathy towards a group that has suffered the most devastating effect of COVID-19 [29, 30] and systematic racism that was laid bare by the Black Lives Matter movement.

COVID-19 pandemic and the accompanying economic collapse dealt a heavy blow on Hispanics as well. Our results appear to suggest that for Hispanics any rise in empathy on account of the devastating effect of the pandemic was counteracted by the rising xenophobia triggered by the pandemic. It is likely that Trump’s anti-immigration rhetoric instilled greater fear and xenophobia towards Hispanics in the wake of COVID-19. It may be that the xenophobic tendencies towards Hispanics were especially salient because of their larger size in the U.S. population. Intergroup and group threat theory postulates that a larger minority (or immigrant) out-group size increases perceptions of threatened in-group interests and anti-minority sentiments [31]. A number of studies have found evidence for this effect [32, 33]. Our findings suggest that Hispanics as the largest immigrant minority group in the country may have suffered on this account.

Lack of an effect towards South Asians is surprising. After all, in the context COVID-triggered xenophobia, South Asians should also be affected. We think that our experiment was more successful in signaling Hispanic than South Asian ethnicity: the manipulation checks show that 83% of the respondents identified Hispanic names correctly versus only 60% did so for South Asians. This likely reflects, as documented in other surveys, that a majority of Americans do not identify Indians and Pakistanis as Asians [34]. The 2016 National Asian American Survey, for instance, found that 41% of Americans did not classify Indians as Asians and 45% did not classify Pakistanis as Asians. The proportion was only 5% for East Asian groups of Chinese, Japanese, and Koreans.

In August 2020, when we conducted our study, the pandemic was raging throughout the United States. We could not measure the impact of COVID-19 directly because of the lack of a counterfactual scenario. We used a vignette experiment priming COVID-19 salience to study its impact on self-reported prejudicial responses towards ethnoracial groups. Arguably, our treatment is moderate because all Americans were impacted by COVID-19. Even respondents in the control group were likely occupied with COVID-related thoughts and concerns when they completed the survey. In that sense, we may have underestimated the true effect of the pandemic on attitudes towards racial/ethnic minorities.

We study attitudes but not actual instances of discrimination. Extant research, however, finds that harboring prejudices towards minority groups prompts discriminatory action [35]. Our finding of rising prejudicial responses towards East Asians and Hispanic co-workers in a hypothetical workplace scenario, alongside reports of rising instances of discrimination and hate crime towards these groups more broadly, provides evidence that these instances are not random events, and that COVID-19 might have increased prejudicial sentiments towards minorities in the workplace.

## Supporting information

S1 File Pandemic and prejudice: Results from a national survey experiment. (DOCX)
