# Supplementary material for: Pandemic and prejudice: Results from a national survey experiment
Source: PLoS One. 2022 Apr 13;17(4):e0265437. doi: 10.1371/journal.pone.0265437 (PMC9007497; doi:10.1371/journal.pone.0265437)
Supplement: S1 File — (DOCX) [file pone.0265437.s001.docx]

**Supplementary Information for**

Pandemic and Prejudice: Results from a National Survey Experiment

Neeraj Kaushal^a,1^, Yao Lu^b^, Xiaoning Huang^c^

Affiliations:

^a^ School of Social Work, Columbia University; New York, United States.

^b^ Department of Sociology, Columbia University; New York, United States.

^C^ Feinberg School of Medicine, Northwestern University; Chicago, United States.

^1^ Corresponding author: Neeraj Kaushal

**Email:** nk464@columbia.edu.

1. Text and Questions for Experiment Treatments


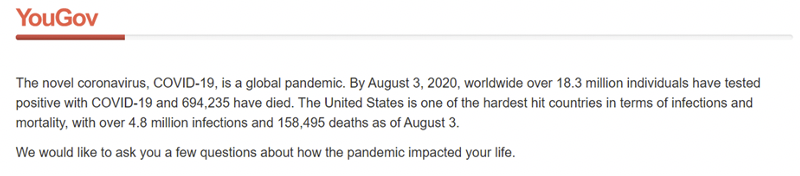


Fig. S1. Covid-19 information treatment (top layer).

Questions:

1) Are you currently working for pay or profit?

2) At any time between March and July 2020, were you doing any work for pay or profit?

3) At any time between March and July 2020, did you telework or work at home for pay because of the coronavirus?

4) At any time between March and July 2020, were you unable to work due to the coronavirus?

5) At any time between March and July 2020, did the terms of your employment change?

6) At any time between March and July 2020, was the employment of your immediate family member(s) affected by coronavirus?

7) At any time between March and July 2020, did you receive a pay from your employer for the hours you did not work?

8) At any time between March and July 2020, did you receive any severance or unemployment benefit?

9) At any time between March and July 2020, did you receive a check from the government under the COVID-19 stimulus package (the CARES Act)?

10) At any time between March and July 2020, did you receive SNAP/food stamps benefits?

11) At any time between March and July 2020, did the coronavirus pandemic prevent you from looking for work?

12) At any time between March and July 2020, were you ever under shelter-in-place, stay-at-home, or safer-at-home orders?

13) Have you, or do you know anyone, who tested positive for COVID-19?

14) Do you know anyone who died from COVID-19? If so, what is your relationship with the deceased?


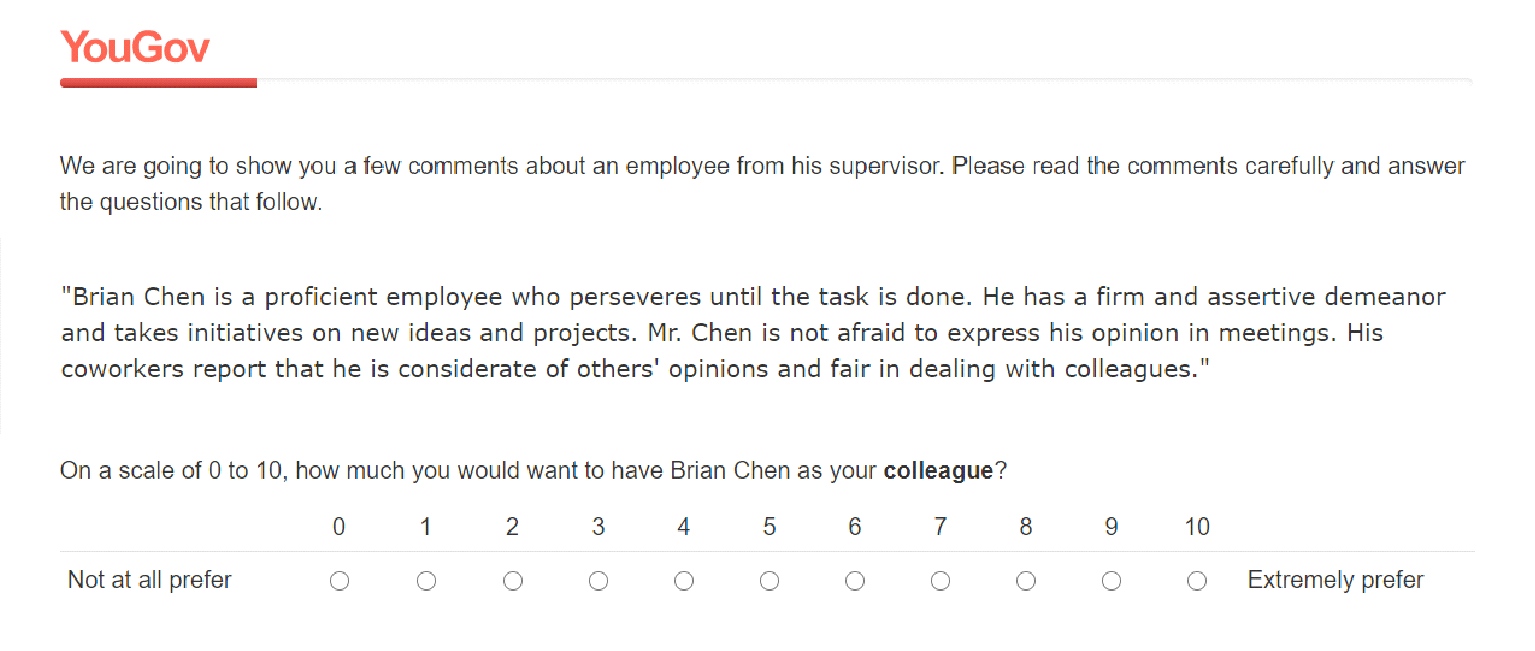


Fig. S2. Racial/ethnic treatment (second layer) vignette coworker experiment.

Additional questions:

1) On a scale of 0 to 10, how much you would want to have Brian Chen as your supervisor?

2) Think of yourself as a supervisor. On a scale of 0 to 10, how much you would want to supervise Brian Chen as your staff member?

Note: The three questions above were presented in random order in the survey.

2. Literature Review

Summary of previous studies on anti-Asian and anti-minority sentiments amid COVID-19

| **Author(s)** | **Year, Title and Journal** | **Data and Methods** | **Main Findings** |
| --- | --- | --- | --- |
| He et al. (1) | 2020. Discrimination and social exclusion in the outbreak of COVID-19. *International Journal of Environmental Research and Public Health* | Online survey to Chinese overseas in 70 countries (n=1,904) fielded in February 2020. | A quarter of respondents reported to have experienced different forms of discrimination. Most respondents who experienced such discrimination were more likely to reside in high-income countries. |
| Reny and Barreto (2) | 2020. Xenophobia in the time of pandemic: othering, anti-Asian attitudes, and COVID-19. *Politics, Groups, and Identities* | Lucid’s Academic Marketplace, Theorem sample (n=4,311) weighted to 2018 American Community Survey targets. Data was collected between March 12 and 15, 2020. | Anti-Asian attitudes are positively associated with greater concern about the disease, more xenophobic behaviors, and preferences for exclusive policies. |
| Ruiz, Horowitz and Tamir (3) | 2020. Many Black and Asian Americans say they have experienced discrimination amid the COVID-19 outbreak. *Pew Research Center* | Pew Research Center’s American Trends Panel, a nationally representative sample of US adults (n=9,654), surveyed online from June 4-10, 2020. | Asian and Black Americans have been more likely than other groups to report negative experiences because of their race or ethnicity since the coronavirus outbreak. Black, Asian adults are more likely than white and Hispanic adults to worry that people will be suspicious if they wear a mask. Three-in-ten or more U.S. adults say racist views about Asian and Black Americans have been more common during the pandemic than before it. |
| Rzymski and Nowicki (4) | 2020. COVID-19-related prejudice toward Asian medical students: a consequence of SARS-CoV-2 fears in Poland. *Journal of infection and public health* | Online survey of Asian medical students in Poland (n=85) conducted in February 2020. | 61.2% of the surveyed Asian students have experienced prejudice in Poland related to the current coronavirus epidemic, and it was more frequently witnessed by those wearing face masks than those who did not (71.2% vs 28.2%). The prejudice was encountered the most on public transportation and on the street. |
| Wu, Qian and Wilkes (5) | 2020. Anti-Asian discrimination and the Asian-white mental health gap during COVID-19. *Ethnic and Racial Studies* | The University of Southern California’s Center for Economic  and Social Research Understanding Coronavirus in America survey, a nationally representative internet panel of American Households (n=7,778, 13 waves) surveyed between March and September 2020. | First, since the onset of the pandemic, Asians (Asian Americans in particular) have experienced higher levels of anxiety or depression than whites. Second, Asian Americans and Asian immigrants are about twice as likely as whites to report having encountered instances of COVID-19- related acute discrimination. Third, experiences of COVID-19-related discrimination increase mental disorders for all Americans. Finally, COVID-19-related discrimination partially explains the disproportionate mental health impact of the pandemic on Asians. |
| Lu et al. (6) | 2021. Priming COVID-19 Salience Increases Prejudice and Discriminatory Intent Against Asians and Hispanics. *Proceedings of the National Academy of Sciences of the United States of America* | Nationally-representative online survey data with an embedded vignette experiment about roommate selection (collected in August 2020; n = 5,000) | Priming COVID-19 salience increased prejudice and discriminatory intent towards East Asian, South Asian, and Hispanic hypothetical room-seekers. Prior social contact with Hispanics mitigated prejudice towards Hispanics. Prior social contact with Asians did not mitigate prejudice towards Asians. |

3. Supplementary Tables

Table S1. COVID-19 information treatment effects on workplace prejudice (sample includes signaled race)

|  |  |  | **How much you would prefer to have the employee just described as your colleague** | | |  | **How much you would prefer to have the employee just described as your supervisor** | | |  | **How much you would prefer to have the employee just described as your staff** | | | |
| --- | --- | --- | --- | --- | --- | --- | --- | --- | --- | --- | --- | --- | --- | --- |
|  | N |  | **Linear  (0-10)** | **Approve  (8/10)** | **Oppose  (0/2)** |  | **Linear  (0-10)** | **Approve  (8/10)** | **Oppose  (0/2)** |  | **Linear  (0-10)** | **Approve  (8/10)** | **Oppose  (0/2)** | |
|  |  |  |  |  |  |  |  |  |  |  |  |  |  | |
| **White** | 528 |  | -0.09 | 0.02 | 0.02 |  | -0.11 | 0.01 | 0.03 |  | -0.08 | 0.05 | 0.03 | |
|  |  |  | (0.18) | (0.04) | (0.01) |  | (0.20) | (0.04) | (0.02) |  | (0.21) | (0.04) | (0.02) | |
|  |  |  |  |  |  |  |  |  |  |  |  |  |  | |
| **Black** | 599 |  | 0.02 | 0.01 | -0.01 |  | 0.04 | 0.03 | 0.01 |  | 0.03 | 0.00 | -0.00 | |
|  |  |  | (0.17) | (0.04) | (0.01) |  | (0.18) | (0.04) | (0.01) |  | (0.17) | (0.04) | (0.01) | |
|  |  |  |  |  |  |  |  |  |  |  |  |  |  | |
| **Hispanic** | 557 |  | -0.49* | -0.05 | 0.04* |  | -0.44* | -0.08* | 0.02 |  | -0.48* | -0.12* | 0.03^†^ | |
|  |  |  | (0.18) | (0.04) | (0.02) |  | (0.19) | (0.04) | (0.02) |  | (0.18) | (0.04) | (0.02) | |
|  |  |  |  |  |  |  |  |  |  |  |  |  |  | |
| **South Asian** | 1,084 |  | -0.12 | -0.04 | 0.00 |  | -0.14 | -0.02 | 0.01 |  | -0.17 | -0.03 | 0.01 | |
|  |  |  | (0.13) | (0.03) | (0.01) |  | (0.14) | (0.03) | (0.01) |  | (0.14) | (0.03) | (0.01) | |
|  |  |  |  |  |  |  |  |  |  |  |  |  |  | |
| **East Asian** | 1,069 |  | -0.34* | -0.04 | 0.03* |  | -0.35* | -0.05^†^ | 0.03* |  | -0.25^†^ | -0.04 | 0.01 | |
|  |  |  | (0.14) | (0.03) | (0.01) |  | (0.14) | (0.03) | (0.01) |  | (0.14) | (0.03) | (0.01) | |
|  |  |  |  |  |  |  |  |  |  |  |  |  |  | |
| Notes: Sample is restricted to working age (19-64) respondents. The samples include respondents of the same race/ethnicity as the hypothetical coworker. Each cell in the table is based on a different regression and provides estimates of COVID information treatment effects. For each question, the first column presents coefficients based on linear regressions. The second and third columns present average marginal effects based on logistic regressions. Row-headings describe hypothetical co-worker's race/ethnicity. Standard errors are in parenthesis.  ^†^ p<0.1 ^*^ p<0.05. | | | | | | | | | | | | | |  |

Table S2. COVID-19 information treatment effects on workplace prejudice (adjusted for pre-treatment characteristics)

|  |  |  | **How much you would prefer to have the employee just described as your colleague** | | |  | **How much you would prefer to have the employee just described as your supervisor** | | |  | **How much you would prefer to have the employee just described as your staff** | | | |
| --- | --- | --- | --- | --- | --- | --- | --- | --- | --- | --- | --- | --- | --- | --- |
|  | N |  | **Linear  (0-10)** | **Approve  (8/10)** | **Oppose  (0/2)** |  | **Linear  (0-10)** | **Approve  (8/10)** | **Oppose  (0/2)** |  | **Linear  (0-10)** | **Approve  (8/10)** | **Oppose  (0/2)** | |
|  |  |  |  |  |  |  |  |  |  |  |  |  |  | |
| **White** | 222 |  | -0.25 | 0.05 | 0.08 |  | -0.31 | -0.02 | 0.04 |  | -0.47 | -0.02 | 0.10*** | |
|  |  |  | (0.28) | (0.05) | (0.06) |  | (0.29) | (0.06) | (0.05) |  | (0.33) | (0.06) | (0.03) | |
|  |  |  |  |  |  |  |  |  |  |  |  |  |  | |
| **Black** | 528 |  | 0.08 | 0.02 | -0.00 |  | 0.07 | 0.03 | 0.01 |  | 0.08 | 0.00 | -0.01 | |
|  |  |  | (0.17) | (0.04) | (0.01) |  | (0.17) | (0.04) | (0.01) |  | (0.16) | (0.04) | (0.02) | |
|  |  |  |  |  |  |  |  |  |  |  |  |  |  | |
| **Hispanic** | 454 |  | -0.71* | -0.11* | 0.05^†^ |  | -0.63* | -0.11* | 0.04^†^ |  | -0.64* | -0.16* | 0.05* | |
|  |  |  | (0.20) | (0.04) | (0.02) |  | (0.20) | (0.04) | (0.02) |  | (0.20) | (0.04) | (0.02) | |
|  |  |  |  |  |  |  |  |  |  |  |  |  |  | |
| **South Asian** | 998 |  | -0.09 | -0.04 | -0.00 |  | -0.10 | -0.02 | 0.01 |  | -0.15 | -0.03 | 0.01 | |
|  |  |  | (0.14) | (0.03) | (0.01) |  | (0.15) | (0.03) | (0.01) |  | (0.14) | (0.03) | (0.01) | |
|  |  |  |  |  |  |  |  |  |  |  |  |  |  | |
| **East Asian** | 1005 |  | -0.33* | -0.05 | 0.02* |  | -0.35* | -0.06^†^ | 0.03* |  | -0.24^†^ | -0.04 | 0.00 | |
|  |  |  | (0.15) | (0.03) | (0.01) |  | (0.15) | (0.03) | (0.02) |  | (0.14) | (0.03) | (0.01) | |
|  |  |  |  |  |  |  |  |  |  |  |  |  |  | |
| Notes: Sample is restricted to working age (19-64) respondents. The samples exclude respondents of the same race/ethnicity as the hypothetical coworker. Each cell in the table is based on a different regression and provides estimates of COVID information treatment effects. For each question, the first column presents coefficients based on linear regressions. The second and third columns present average marginal effects based on logistic regressions. Row-headings describe hypothetical co-worker's race/ethnicity. All models control for the order of questions asked, respondent's age, gender, race, education, marital status, family income, political party affiliation, log value of population in county of residency, and region of residency. Standard errors are in parenthesis and clustered at county level. ^†^ p<0.1 ^*^ p<0.05. | | | | | | | | | | | | | |  |

Table S3. COVID-19 information treatment effects on workplace prejudice (full sample)

|  |  |  | **How much you would prefer to have the employee just described as your colleague** | | |  | **How much you would prefer to have the employee just described as your supervisor** | | |  | **How much you would prefer to have the employee just described as your staff** | | | |
| --- | --- | --- | --- | --- | --- | --- | --- | --- | --- | --- | --- | --- | --- | --- |
|  | N |  | **Linear  (0-10)** | **Approve  (8/10)** | **Oppose  (0/2)** |  | **Linear  (0-10)** | **Approve  (8/10)** | **Oppose  (0/2)** |  | **Linear  (0-10)** | **Approve  (8/10)** | **Oppose  (0/2)** | |
|  |  |  |  |  |  |  |  |  |  |  |  |  |  | |
| **White** | 676 |  | -0.18 | -0.01 | 0.01 |  | -0.23 | -0.03 | 0.03^†^ |  | -0.15 | 0.01 | 0.02 | |
|  |  |  | (0.16) | (0.03) | (0.01) |  | (0.17) | (0.04) | (0.02) |  | (0.17) | (0.04) | (0.02) | |
|  |  |  |  |  |  |  |  |  |  |  |  |  |  | |
| **Black** | 735 |  | 0.01 | 0.02 | -0.00 |  | 0.06 | 0.03 | 0.01 |  | 0.07 | 0.02 | -0.00 | |
|  |  |  | (0.15) | (0.03) | (0.01) |  | (0.16) | (0.03) | (0.01) |  | (0.15) | (0.03) | (0.01) | |
|  |  |  |  |  |  |  |  |  |  |  |  |  |  | |
| **Hispanic** | 734 |  | -0.26^†^ | -0.00 | 0.04* |  | -0.17 | -0.02 | 0.02 |  | -0.29^†^ | -0.09* | 0.03* | |
|  |  |  | (0.16) | (0.03) | (0.02) |  | (0.16) | (0.03) | (0.01) |  | (0.15) | (0.03) | (0.02) | |
|  |  |  |  |  |  |  |  |  |  |  |  |  |  | |
| **South Asian** | 1438 |  | -0.03 | -0.02 | -0.00 |  | -0.03 | -0.00 | 0.01 |  | -0.06 | -0.01 | 0.01 | |
|  |  |  | (0.11) | (0.02) | (0.01) |  | (0.12) | (0.03) | (0.01) |  | (0.12) | (0.02) | (0.01) | |
|  |  |  |  |  |  |  |  |  |  |  |  |  |  | |
| **East Asian** | 1417 |  | -0.27* | -0.03 | 0.03* |  | -0.27* | -0.03 | 0.04* |  | -0.22^†^ | -0.03 | 0.01 | |
|  |  |  | (0.12) | (0.02) | (0.01) |  | (0.12) | (0.03) | (0.01) |  | (0.12) | (0.02) | (0.01) | |
|  |  |  |  |  |  |  |  |  |  |  |  |  |  | |
| Notes: The samples include respondents of the same race/ethnicity as the hypothetical coworker. Each cell in the table is based on a different regression and provides estimates of COVID information treatment effects. For each question, the first column presents coefficients based on linear regressions. The second and third columns present average marginal effects based on logistic regressions. Row-headings describe hypothetical co-worker's race/ethnicity. Standard errors are in parenthesis. ^†^ p<0.1 ^*^ p<0.05. | | | | | | | | | | | | | |  |

Table S4. Summary statistics of pre-treatment characteristics

|  |  | **Treatment Group**  **N=1,915** | |  | **Control Group**  **N=1,922** | |
| --- | --- | --- | --- | --- | --- | --- |
| **Variables** |  | **Mean** | **SD** |  | **Mean** | **SD** |
| **Age** |  |  |  |  |  |  |
| **19-35** |  | 0.35 | 0.48 |  | 0.35 | 0.48 |
| **36-50** |  | 0.31 | 0.46 |  | 0.29 | 0.46 |
| **51-64** |  | 0.35 | 0.48 |  | 0.35 | 0.48 |
| **Gender (Female)** |  | 0.53 | 0.50 |  | 0.53 | 0.50 |
| **Race** |  |  |  |  |  |  |
| **White** |  | 0.58 | 0.49 |  | 0.61 | 0.49 |
| **Black** |  | 0.13 | 0.34 |  | 0.12 | 0.33 |
| **Hispanic** |  | 0.19 | 0.39 |  | 0.17 | 0.37 |
| **Asian and others** |  | 0.10 | 0.30 |  | 0.10 | 0.30 |
| **Education** |  |  |  |  |  |  |
| **High school or less** |  | 0.33 | 0.47 |  | 0.33 | 0.47 |
| **Some college** |  | 0.33 | 0.47 |  | 0.35 | 0.48 |
| **College graduates** |  | 0.34 | 0.47 |  | 0.32 | 0.47 |
| **Family income (in USD)** |  |  |  |  |  |  |
| **<=29999** |  | 0.26 | 0.44 |  | 0.25 | 0.43 |
| **30000-59999** |  | 0.24 | 0.43 |  | 0.27 | 0.44 |
| **>=60000** |  | 0.38 | 0.49 |  | 0.38 | 0.48 |
| **Prefer not to say** |  | 0.11 | 0.32 |  | 0.11 | 0.31 |
| **Political parties** |  |  |  |  |  |  |
| **Republican** |  | 0.39 | 0.49 |  | 0.38 | 0.48 |
| **Independent/Others** |  | 0.38 | 0.48 |  | 0.40 | 0.49 |
| **Democrat** |  | 0.24 | 0.43 |  | 0.23 | 0.42 |
| **Regions** |  |  |  |  |  |  |
| **Northeast** |  | 0.18 | 0.38 |  | 0.18 | 0.39 |
| **Midwest** |  | 0.21 | 0.40 |  | 0.20 | 0.40 |
| **South** |  | 0.38 | 0.49 |  | 0.38 | 0.48 |
| **West** |  | 0.24 | 0.43 |  | 0.24 | 0.43 |
|  |  |  |  |  |  |  |
| Note: Sample is restricted to working age (19-64) respondents. | | | | | | |

Table S5. Names used in the vignette experiment

| Race/Ethnicity | Names |
| --- | --- |
| White | Matthew McGrath |
| Black | Tyrone Washington |
| Hispanic | Fernando Vasquez |
| South Asian | Michael Patil / Aditya Patel |
| East Asian | Brian Chen / Peng Chen |

**Table S6.** Full results for linear regression models: Response towards hypothetical colleague

|  | **White** | **Black** | **Hispanic** | **South Asian** | **East Asian** |
| --- | --- | --- | --- | --- | --- |
|  |  |  |  |  |  |
| **COVID Treatment** | -0.25 | 0.08 | -0.71* | -0.09 | -0.33* |
|  | (0.28) | (0.17) | (0.20) | (0.14) | (0.15) |
| **Age:** |  |  |  |  |  |
| **Age: 19-35** | ref. | ref. | ref. | ref. | ref. |
| **Age: 36-50** | -0.25 | -0.13 | -0.09 | 0.07 | 0.18 |
|  | (0.40) | (0.22) | (0.25) | (0.18) | (0.19) |
| **Age: 51-64** | -0.09 | -0.09 | 0.24 | 0.40* | 0.15 |
|  | (0.42) | (0.23) | (0.21) | (0.18) | (0.18) |
|  |  |  |  |  |  |
| **female** | 0.47 | 0.35† | 0.40* | 0.02 | -0.21 |
|  | (0.29) | (0.18) | (0.20) | (0.14) | (0.15) |
| **Race:** |  |  |  |  |  |
| **White** |  | ref. | ref. | ref. | ref. |
| **Black** | ref. |  | -0.17 | -0.60* | -0.33 |
|  |  |  | (0.36) | (0.23) | (0.27) |
| **Hispanic** | 0.09 | -0.42† |  | -0.52* | -0.17 |
|  | (0.44) | (0.23) |  | (0.21) | (0.19) |
| **Asian and Others** | 0.26 | -0.25 | -0.67* | -0.60† | -0.06 |
|  | (0.51) | (0.29) | (0.34) | (0.35) | (0.33) |
| **Education:** |  |  |  |  |  |
| **High school or less** | ref. | ref. | ref. | ref. | ref. |
| **Some college** | 0.71† | 0.41† | 0.84* | 0.64* | 0.53* |
|  | (0.38) | (0.24) | (0.27) | (0.17) | (0.19) |
| **College graduates** | 0.36 | 0.54* | 0.93* | 0.53* | 0.40* |
|  | (0.50) | (0.21) | (0.32) | (0.18) | (0.18) |
|  |  |  |  |  |  |
| **Married/In union** | 0.09 | 0.15 | 0.48* | 0.09 | 0.34* |
|  | (0.33) | (0.20) | (0.21) | (0.16) | (0.17) |
| **Income:** |  |  |  |  |  |
| **Income<29999** | ref. | ref. | ref. | ref. | ref. |
| **30000-59999** | 0.81* | 0.51† | 0.19 | 0.15 | 0.27 |
|  | (0.38) | (0.28) | (0.32) | (0.22) | (0.21) |
| **Income>=60000** | 0.62 | 0.83* | -0.28 | 0.17 | 0.50* |
|  | (0.50) | (0.26) | (0.35) | (0.22) | (0.20) |
| **Prefer not to say** | -0.99 | -0.12 | -0.49 | -0.20 | -0.29 |
|  | (0.79) | (0.40) | (0.37) | (0.30) | (0.29) |
| **Party:** |  |  |  |  |  |
| **Ind/Others** | ref. | ref. | ref. | ref. | ref. |
| **Democrat** | 0.88* | 0.26 | -0.10 | 0.41* | 0.42* |
|  | (0.35) | (0.20) | (0.25) | (0.18) | (0.18) |
| **Republican** | 1.28* | -0.51* | -0.27 | 0.08 | 0.12 |
|  | (0.44) | (0.23) | (0.27) | (0.22) | (0.20) |
|  |  |  |  |  |  |
| **Log county population 2019** | -0.03 | -0.10† | 0.16* | -0.01 | 0.01 |
|  | (0.09) | (0.06) | (0.07) | (0.05) | (0.06) |
| **Region:** |  |  |  |  |  |
| **Northeast** | ref. | ref. | ref. | ref. | ref. |
| **Midwest** | 0.97 | 0.32 | -0.09 | -0.01 | 0.11 |
|  | (0.68) | (0.28) | (0.32) | (0.23) | (0.23) |
| **South** | 0.56 | 0.69* | 0.03 | 0.00 | 0.01 |
|  | (0.52) | (0.26) | (0.29) | (0.19) | (0.21) |
| **West** | 0.60 | 0.44† | 0.20 | 0.05 | 0.03 |
|  | (0.53) | (0.26) | (0.33) | (0.21) | (0.21) |
|  |  |  |  |  |  |
| **Constant** | 5.68* | 8.49* | 5.36* | 7.09* | 7.44* |
|  | (1.32) | (0.95) | (1.01) | (0.73) | (0.77) |
|  |  |  |  |  |  |
| **Observations** | 222 | 528 | 454 | 998 | 1005 |
|  |  |  |  |  |  |

Notes: Sample is restricted to working age (19-64) respondents. The samples exclude respondents of the same race/ethnicity as the hypothetical coworker. Each column in the table is based on a linear regression and provides estimates of COVID information treatment effects on respondents’ attitudes towards hypothetical colleagues. Column-headings describe hypothetical co-worker's race/ethnicity. In addition to the variables in the table, all models control for the order of questions asked. Standard errors are in parenthesis and clustered at county level. ^†^ p<0.1 ^*^ p<0.05.

Table S7. Heterogenous effects across age and gender groups

|  |  | **Hispanic Coworker** | | |  | **East Asian Coworker** | | |
| --- | --- | --- | --- | --- | --- | --- | --- | --- |
|  |  | **Colleague** | **Supervisor** | **Staff** |  | **Colleague** | **Supervisor** | **Staff** |
| **Panel 1: Working age sample** |  |  |  |  |  |  |  |  |
| **COVID information treatment** |  | -0.75* | -0.63* | -0.69* |  | -0.37* | -0.38* | -0.25 |
|  |  | (0.32) | (0.31) | (0.30) |  | (0.18) | (0.19) | (0.17) |
|  |  |  |  |  |  |  |  |  |
| **Female** |  | 0.39^†^ | 0.48^†^ | 0.19 |  | -0.19 | -0.09 | -0.37* |
|  |  | (0.23) | (0.25) | (0.23) |  | (0.19) | (0.19) | (0.18) |
|  |  |  |  |  |  |  |  |  |
| **Treatment × Female** |  | 0.06 | -0.05 | 0.05 |  | 0.04 | 0.02 | -0.01 |
|  |  | (0.42) | (0.42) | (0.39) |  | (0.28) | (0.28) | (0.28) |
|  |  |  |  |  |  |  |  |  |
| **Panel 2: No age restriction** |  |  |  |  |  |  |  |  |
| **COVID information treatment** |  | -0.61* | -0.55* | -0.70* |  | -0.39* | -0.37* | -0.34* |
|  |  | (0.22) | (0.22) | (0.22) |  | (0.17) | (0.17) | (0.16) |
|  |  |  |  |  |  |  |  |  |
| **Age>54** |  | -0.47 | -0.46 | -0.34 |  | -0.49^†^ | -0.35 | -0.18 |
|  |  | (0.32) | (0.33) | (0.30) |  | (0.26) | (0.27) | (0.25) |
|  |  |  |  |  |  |  |  |  |
| **Treatment × Age>54** |  | 0.39 | 0.50^†^ | 0.61* |  | 0.28 | 0.22 | 0.33 |
|  |  | (0.29) | (0.30) | (0.30) |  | (0.26) | (0.26) | (0.24) |
|  |  |  |  |  |  |  |  |  |
| **Panel 2: No age restriction** |  |  |  |  |  |  |  |  |
| **COVID information treatment** |  | -0.74* | -0.65* | -0.68* |  | -0.34* | -0.36* | -0.24^†^ |
|  |  | (0.20) | (0.20) | (0.20) |  | (0.15) | (0.15) | (0.14) |
|  |  |  |  |  |  |  |  |  |
| **Age>64** |  | -0.36 | -0.41 | -0.22 |  | -0.29 | -0.21 | 0.01 |
|  |  | (0.32) | (0.35) | (0.33) |  | (0.21) | (0.22) | (0.22) |
|  |  |  |  |  |  |  |  |  |
| **Treatment × Age>64** |  | 1.15* | 1.23* | 0.98* |  | 0.33 | 0.37 | 0.18 |
|  |  | (0.35) | (0.39) | (0.35) |  | (0.27) | (0.29) | (0.25) |
|  |  |  |  |  |  |  |  |  |
| Notes: Panel 1is restricted to working age (19-64) respondents. Panel 2 and 3 include all adults in the sample. The samples exclude respondents of the same race/ethnicity as the hypothetical coworker. Each column within each panel in the table is based on a linear regression. Outcomes on coworker preferences are measured on a scale of 0 to 10 (higher number indicating greater preference). All regression models control for the order of questions asked, respondent's age, gender, race, education, marital status, family income, log value of population in county of residency, and region of residency. The top-row column headings list the race/ethnicity of the hypothetical co-worker in the vignette. Standard errors are in parenthesis and clustered at county level. ^†^ p<0.1 ^†^ p<0.05. | | | | | | | | |

**4. SI References**

1. He J, He L, Zhou W, Nie X, He M. Discrimination and social exclusion in the outbreak of COVID-19. International Journal of Environmental Research and Public Health. 2020;17(8):2933.

2. Reny TT, Barreto MA. Xenophobia in the time of pandemic: othering, anti-Asian attitudes, and COVID-19. Politics, Groups, and Identities. 2020:1-24.

3. Ruiz NG, Horowitz J, Tamir C. Many Black and Asian Americans say they have experienced discrimination amid the COVID-19 outbreak. Pew Research Center; 2020.

4. Rzymski P, Nowicki M. COVID-19-related prejudice toward Asian medical students: a consequence of SARS-CoV-2 fears in Poland. Journal of infection and public health. 2020;13(6):873-6.

5. Wu C, Qian Y, Wilkes R. Anti-Asian discrimination and the Asian-white mental health gap during COVID-19. Ethnic and Racial Studies. 2020:1-17.

6. Lu Y, Kaushal N, Huang X, Gaddis MS. Priming COVID-19 Salience Increases Prejudice and Discriminatory Intent Against Asians and Hispanics. Proceedings of the National Academy of Sciences of the United States of America. 2021.

7. Bailey ZD, Moon JR. Racism and the political economy of COVID-19: will we continue to resurrect the past? Journal of Health Politics, Policy and Law. 2020;45(6):937-50.

8. Parilla J, Bouchet M. Which US communities are most affected by Chinese, EU, and NAFTA retaliatory tariffs? Brookings Institutions. 2018(October 2018).
